# What We Owe to Experiments on Animals

**Published:** 1904-01-30

**Authors:** Stephen Paget


### HOSPITAL CLINICS AND MEDICAL PROGRESS.

### WHAT WE OWE TO EXPERIMENTS ON ANIMALS.

By STEPHEN PAGET.

### II.—PATHOLOGY, BACTERIOLOGY, AND THERAPEUTICS.

(*Continued from page* 290.)

#### VIII.—PLAGUE.

As with cholera, so with plague, Haffkine's name stands first. The plague bacillus was discovered by Kitasato and Yersin, working independently, in 1894; the immunisation of animals was proved and formulated in 1896, and the first person inoculated with Haffkine's anti-plague fluid was himself, January 10, 1897: then Surgeon-Colonel Hatch and others on the staff of the Grant Medical College, Bombay. Let Haffkine here tell his own story (*Lecture at Poona*, 1901):—

"In a short time, a number of the most authoritative physicians in Bombay, European and native, official medical officers and private practitioners, submitted themselves for inoculation (including Surgeon-General Bainbridge, Head of the Medical Service of the Presidency, and Surgeon-General Harvey, Director-General of the Indian Medical Service). It was the example of these gentlemen that encouraged thousands of people, rich and poor, in Bombay and elsewhere, to come forward for inoculation. Thus his Excellency the Viceroy thought it right to tell you here, in Poona, that previous to his starting for the plague-stricken districts he and his staff had also undergone the prophylactic inoculation."

On January 23, 1897, less than a fortnight after Haffkine's self-inoculation, plague attacked the Byculla House of Correction, Bombay. By January 30, there had been 14 cases, with 7 deaths. That afternoon, January 30, 152 prisoners were inoculated, and 172 were left without inoculation. A week later, the plague ceased. The 152 had 1 case, which got well; the 172 had 7 cases, with 2 deaths. About the same time, plague attacked Mora, a village near Bombay, with a population of less than 1,000. Of these, 429 were inoculated, and the rest (let us say 571) were left without inoculation. The 429 had 7 cases, which all recovered: the 571 had 26 cases, with 24 deaths.

This preventive treatment is not yet seven years old. It had hardly got outside the doors of the laboratory before the whole world was astir over it. In 1899 Spain, Italy, and Turkey were begging the Pasteur Institute (Paris) for supplies of the fluid, the Pasteur Institute was hugging a store for home use, London was asking the Indian Government whether 50,000 doses a week could be consigned to her in case of need, Russia was trying to get a consignment for Port Arthur, Portugal wanted enough to inoculate Mozambique, and the Bombay laboratory was fitting itself to turn out 10,000 doses daily, and was in need of enlargement to do more. (Contrast, with this world-faith, the slow welcome that India gave to ordinary vaccination. It was a quarter of a century before the number of vaccinations in Calcutta reached an average of 2,000 annually.) As for India herself, and the rush of the work there in 1897–1898, only one man could do justice to it, and that is Mr. Rudyard Kipling: he ought to write the story, how they fought the plague at Hubli and elsewhere, with their lives in their hands.

The Report of the Plague Commission (1901), a monumental blue book of 450 pages, deals with that magnificent story in a very different way; it sifts and re-sifts every particle of evidence and every list of results, and is terribly hard. Still, enough is left to show that the bacteriologists are working on the right lines. Take the following instances:—

1. *Daman, Portuguese territory, March–May*, 1897. (i.) Among the Parsee community in Daman, 306 in all, 277 were inoculated, and only 29 were left without inoculation. Plague picked out four cases from this mere handful of the non-inoculated=13·8 per cent., and one from all the inoculated=0·36 per cent. (ii.) Major Lyons, President of the Bombay Plague Committee, made a house-to-house visitation of 89 houses. In 62 of them, inoculated and non-inoculated were living side by side. Among 123 non-inoculated, 38 died=30·9 per cent. Among 382 inoculated, 36 died=9·4 per cent.

2. *Lanauli, a small hill-station, April-Sept.* 1897.—The Commissioners do not accept as accurate the statistics from this little town; but they are agreed that the inoculations "exerted a distinct preventive effect;" and they practically admit Major Baker's evidence—"In the place where inoculation had been made use of, the town was thriving and full of people; and the other part of the town was absolutely empty. One side had plague, and the other had none."

3. *Kirki, near Poona; Artillery cantonment*, 1897.—The Commissioners practically find no fault with these statistics. Among 671 inoculated, there were 32 cases, with 17 deaths=2·5 per cent. Among 859 non-inoculated, there were 143 cases, with 98 deaths =11·4 per cent. "Here," says Surg.-Maj. Bannerman, Superintendent of the Bombay Plague Laboratory, "is seen a body of people, divided into two groups by the fact that one had undergone inoculation and the other not, *but differing in no other way. Seeing the absolute similarity of conditions*, the 671 inoculated should have had proportionately 112 cases and 77 deaths, if they had remained as susceptible to the disease as their uninoculated brothers, sisters, parents, wives, husbands, children; but, instead of that, they had only 32 cases and 17 deaths. The death-rate would doubtless have been still further reduced but for the fact that *a very much weakened vaccine* had to be used, owing to the demand having got beyond the resources of the laboratory at that time."

4. *Belgaum, Southern India*, 1897–1898. The Commissioners do not admit the accuracy of the general statistics. But they admit, as substantially exact, the Army cases; and, as absolutely exact, Major Forman's cases. (i.) The Army cases occurred in the 26th Madras Infantry. Plague attacked the regiment on November 12th, 1897, and by December 23rd there had been 78 cases, with 49 deaths. During the next week 1,665 persons were inoculated. The epidemic in the city was at its height in January 1898, and lasted to May, 1898. *All these months, out of all the 1,665 inoculated, though they went just where they liked, only two were attacked, and both of them recovered.* In July, the epidemic broke out again in Belgaum, and inoculations were again made throughout the regiment, 1,801 in all. *In the first epidemic, before inoculation, 78 cases occurred, and 2 after it. In the second, and much more severe, epidemic, though the sanitary measures adopted in both epidemics were similar, only 12 cases occurred.* (ii.) Major Forman's cases were (α) his own servants with their families; 31 persons in all. Of 28 inoculated, none were attacked; of 3 not inoculated, 2 are said to have died of plague, and 1 certainly did. (β) The hospital servants, 90 persons in all. Of 87 inoculated, 1 died of plague; of 3 not inoculated, 2 died of plague.

5. *Umarkhadi Jail, Bombay.*—Plague attacked the jail on the last day of 1897, and 3 prisoners died. Next day all were paraded, 402 in all, and were divided into two equal groups of 201 each. Two prisoners refused inoculation: thus, 199 were inoculated, and 203 left without inoculation. The two groups had *the same food and drink, the same hours of work and rest, and the same accommodation.* The plague lasted till March, 1898. The Commissioners note "the important fact that, *during the whole period of the outbreak, the number of attacks among the inoculated was only one-third of the number among the uninoculated; and that the disease among the inoculated was remarkably mild, resembling mumps more than plague, though the cases among the uninoculated were of average severity.*"

6. *Undhera, a village near Baroda, Jan.–April,* 1898. The figures here are small, but of great interest. Plague attacked the village in January, 1898. Of 1,029 persons, 76 were dead of it by February 12. *On that day the whole of the inhabitants were called out, house by house, and the half of each household was inoculated.* On April 4, a house-to-house investigation was made. The figures are as follows: Population on February 12, 1,029—76=953. Inoculated, 513; not inoculated, 400. The 513 had 8 cases with 3 deaths = 0·6 per cent. The 440 had 28 cases with 27 deaths = 6·0 per cent.

7. *Hubli,* 50,000 *inhabitants, May–Sept.,* 1898. The story of the long hand-to-hand fight with death at Hubli, and the strength and endurance of Leumann and those with him, demand a poet; and Tennyson would have loved the subject. Leumann himself only hints at a few of the many appalling difficulties of the work—"They have presented themselves by the hundred, at all times of the day, to be inoculated. . . . Not only were the poorer, dirtier, lower-caste people the first to be persuaded to receive inoculation, but I made it my personal and special duty to work amongst them. . . . So few police (and those none too good) to help me; an inadequate British staff, a municipality so bankrupt that it could not, apparently, afford enough blankets for the patients in the plague hospitals." In less than five months, 78,000 inoculations. The whole history of the Mutiny, and all the little patriotic books of to-day, *Fights for the Flag* and all the rest of them, contain nothing to beat this story of Hubli. The Commissioners chilled it down from a miracle to a fact; but they, even they, seem to feel here that statistics are not everything:—"It is quite clear that a very large number of lives must have been saved in Hubli by inoculations during the whole course of the epidemic there. Moreover, we may note that an arithmetical estimate is not the only criterion by which we can appreciate the value of inoculations. And in Hubli their value is approved by the consensus of opinions of officers who have seen probably far more of this process and its results in practice than any other persons in India, and who, having every facility for forming a sound judgment as to its effect where plague was really virulent, are satisfied as to its great value."

---

There is no room here for more instances. One disaster has occurred, most unhappily, from the use of some fluid that was contaminated. Also the standardisation of the fluid, in 1898, was not all that it ought to be. The *curative* use of Haffkine's fluid, or of some similar fluid, for patients already stricken by plague, has given results that are hopeful, but nothing more. "In plague, as in other infectious diseases," say the Commissioners, "it is the only method which holds forth a prospect of ultimate success." Some sort of account of these and other matters is put in the book "Experiments on Animals"; and the summary given there may be quoted here. "Of course, there is not, and perhaps there never will be, a national acceptance and adoption of this preventive treatment through the length and breadth of India. It does not work miracles; it is an uncomfortable process to submit to; privileges must be offered with it, or the native will often prefer to take his chance; the protection is of uncertain duration; all sorts of lies are told about it, partly by anti-vivisectionist writers, partly by native political agitators, partly by the *hakims.* Now and for many years to come, preventive inoculation must fall into line with other world-wide ways of fighting plague—quarantine, notification, isolation, all sanitary measures, destruction of rats —*le rat, le génie de la peste*—evacuation of infected towns, disinfection or unroofing of infected houses. Happily, this is just what it does. That admirable paper, the *Indian Medical Gazette* (September, 1901) has put this fact very simply: 'No one ever imagined that inoculation was the *only* means of fighting plague. Its great value consists in its immediate application. To sanitate, ventilate, and practically rebuild a town or village, takes time; and in the meantime thousands die.' For sudden outbursts of plague—since rats (or, more probably, their fleas) are one chief source of infection, and notification is fundamentally abhorrent to native custom, and evacuation may ruin trade, or spread infection, or be impossible by reason of the rains—since 'East is East, and West is West'—it is not always possible to provide, for an Indian village smitten by plague, the excellent arrangements of the Western world. In all such cases, and in all cases of epidemic plague within narrow limits, as in jails, barracks, mills, and the like centres of human life; and in all inner communities, such as the Parsee community at Daman, or the Jewish community at Aden—by every test of this kind, the saving power of preventive inoculation has been proved, again and again, past all doubt. As for those larger death-traps, Hubli, Dhárwár, and the rest of them, here, though the statistics are inexact, we have the word of the men and women themselves who stood between the dead and the living, and the plague was stayed."

(*To be continued.*)

